# Phenology of reproductive condition varies with age and spring weather conditions in male *Myotis daubentonii* and *M. nattereri* (Chiroptera: Vespertilionidae)

**DOI:** 10.1038/s41598-020-63538-y

**Published:** 2020-04-20

**Authors:** Danielle M. Linton, David W. Macdonald

**Affiliations:** University of Oxford, Department of Zoology, Wildlife Conservation Research Unit (WildCRU), The Recanati-Kaplan Centre, Tubney House, Abingdon Road, Tubney, OX13 5QL UK

**Keywords:** Evolutionary ecology, Zoology

## Abstract

We examine the extent to which intrinsic and extrinsic factors influence reproductive phenology in male bats at the population level. Using data from thirteen breeding seasons (2006–2018), encompassing the reproductive histories of 1546 *Myotis daubentonii* and 530 *M. nattereri* males, we compare rates of sexual maturation and the temporal distribution of phases of spermatogenesis between juvenile (born that season) and adult (born in previous seasons) males. We found that (i) higher proportions of *M. daubentonii* (50.81%) than *M. nattereri* (12.85%) became sexually mature as juveniles, (ii) the proportion of juveniles in reproductive condition per annum was influenced by spring weather conditions, (iii) in both species males that reached puberty as juveniles had higher body mass, on average, than immature juveniles, (iv) older males (aged ≥4 years old) commenced spermatogenesis earlier than young adult males (aged 1–3 years old), whilst juveniles that commenced spermatogenesis did so later in the year than adults, in both species, and (v) *M. daubentonii* commenced and completed spermatogenesis earlier than *M. nattereri* in the equivalent age class. Our findings suggest that selection pressure exists for early mating readiness and synchronisation with female receptivity.

## Introduction

Seasonal variation in climate and resource availability (e.g. prey abundance) imposes energetic constraints on the timing of reproduction for many organisms in both temperate and tropical environments^1,2^. Previous studies have confirmed the existence of seasonal breeding cycles in bats from several genera, occupying diverse habitats and ecological niches^3^. Flexibility in the frequency, timing, and characteristics (e.g. whether or not prolonged sperm storage occurs in either sex) of breeding cycles are even known to occur across the range of some widely distributed species in response to local conditions^4^. At temperate latitudes the reproductive cycle of hibernating bat species is interrupted by winter^5^. The mating season commences in late summer and mating activity peaks during autumn^6^, but copulation also occurs (during periods of arousal for one or both parties) throughout hibernation^7,8^, and some mating effort may resume during spring (to varying extents in different species)^9^.

Prolonged sperm storage can occur in bats of either sex, enabling separation of spermatogenesis and mating effort for males^5^, and an interval (often of several months duration) between copulation and conception in breeding females^10^. Sperm storage over winter enables inseminated females to commence gestation as early as possible on emergence from hibernation (i.e. without using limited energy reserves or spending time on mating activities in spring)^11^. Earlier parturition, given favourable conditions, can increase reproductive success and survival rates of both mother and offspring^12–14^. Prolonged storage of viable sperm in male bats has enabled a unique asynchrony to develop between spermatogenesis and the functioning of accessory sex organs and libido^5^. Spermatogenesis commences in spring in adult males in temperate zones and is completed during the peak of food availability and favourable climatic conditions during summer. Sperm then transfer to the caudae epididymides and mating readiness occurs in synchrony with females from late summer onwards^5,15,16^.

Testicular size and manifest engorged epididymides correspond with histological examination of seminiferous tubules during spermatogenesis, and of stored epididymal spermatozoa, respectively^5^. External physical examination and visual assessment of testes and epididymides enlargement therefore provide robust and less invasive measures of reproductive condition for monitoring seasonal patterns of reproductive condition in wild populations^6^.

Juvenile males can achieve sexual maturity during the season of their birth^17–21^. Birth timing is indirectly influenced by spring weather conditions affecting the duration of gestation and parturition dates^14,22^. Whether the proportion of juvenile males becoming sexually mature varies between breeding seasons has not previously been ascertained.

Previous studies on the phenology of reproductive condition in wild male bats have focused on opportunistic sampling during autumn swarming activities at hibernation sites^6^. To our knowledge this is the first study with repeat sampling of known individuals from their summer habitat, following male juveniles from volancy through to sexual maturation, including comparison of reproductive phenology between juveniles, young adults (aged 1–3 years old) and older males (aged ≥4 years old).

Based on our observations that *M. daubentonii* are born earlier than *M. nattereri* within our study system^14^, we predict that:

(i) higher proportions of *M. daubentonii* than *M. nattereri* males will reach sexual maturity as juveniles,
(ii) higher proportions of juvenile males will come into reproductive condition during breeding seasons with favourable spring weather conditions (when parturition dates are earlier),
(iii) spermatogenesis will commence and be completed earlier in *M. daubentonii* compared to *M. nattereri* (we postulate this enables males to synchronise with the reproductive phenology of females, with mating activities commencing as soon as females become receptive at the end of the maternity period).
  Based on longitudinal observations of changes in male body mass and spermatogenesis with age, we also test whether:
(iv) juveniles achieving puberty have higher body mass, on average, than immature juveniles, and adult non-breeders have lower body mass on average compared to adults that undergo spermatogenesis.
(v) spermatogenesis occurs earlier in older males (aged ≥4 years old) compared to young adult males (aged 1–3 years old), and juveniles that reach puberty commence spermatogenesis later than adult males.

## Results

### Age at sexual maturity varies between species

**Figure 1:**
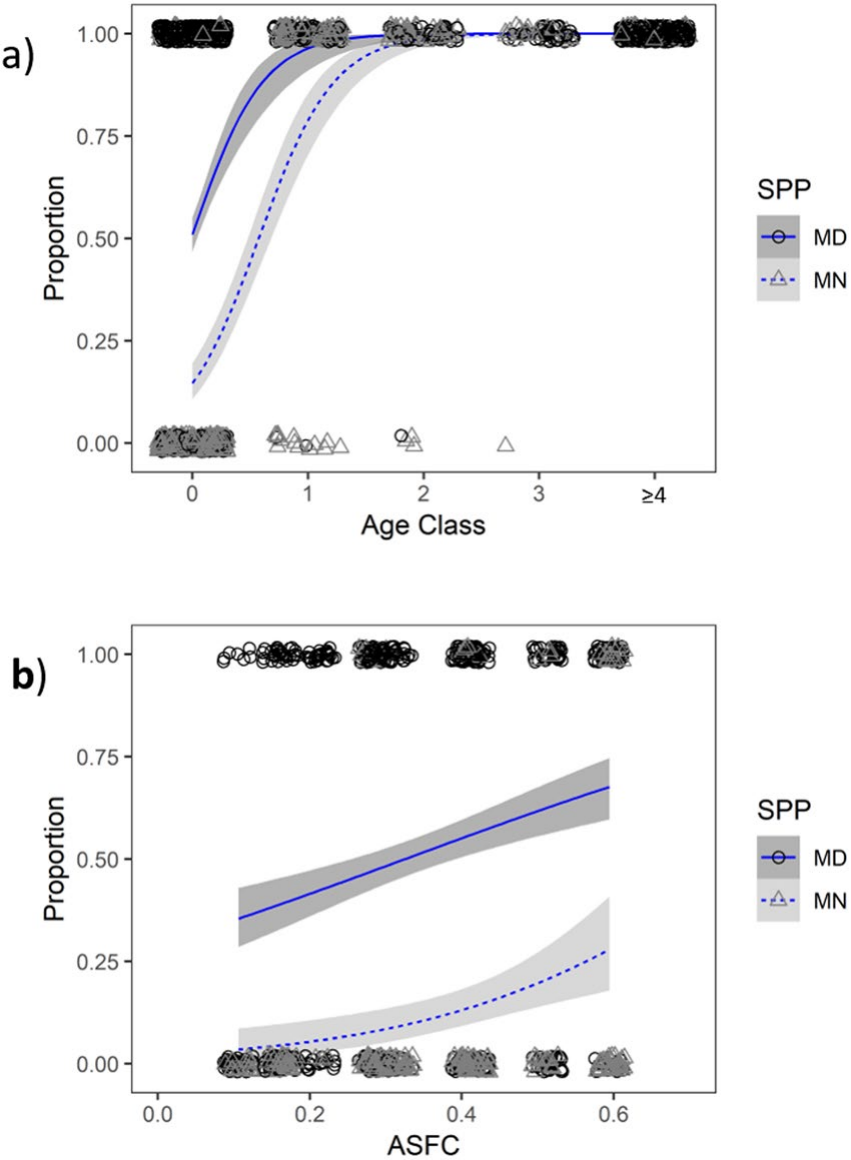
(**a**) Proportion of male *Myotis daubentonii* (MD) and *M. nattereri* (MN) in reproductive condition per age class (0, 1, 2, 3 and ≥4 years old). (**b**) Influence of spring weather conditions (ASFC) on the proportion of juvenile male *Myotis daubentonii* (MD) and *M. nattereri* (MN) in reproductive condition.

As predicted, greater proportions of juvenile male *M. daubentonii* than *M. nattereri* became sexually mature during their birth year (at approximately 2–5 months old) (see Fig. 1a and Model I in Table 1). 282/555 (50.81%) juvenile male *M. daubentonii* reached puberty (reproductive condition B, C or D), the other 273/555 (49.19%) were still sexually immature (non-reproductive condition A) during September or October in the year of their birth. In marked contrast, only 32/249 (12.85%) juvenile male *M. nattereri* were found in reproductive condition. Even if ‘unknown’ (minimum age = 0) *M. nattereri* males are included (which is likely to produce an overestimate as this category comprises a mixture of advanced juveniles and young adults) only 46/259 (17.76%) male *M. nattereri* of juvenile and ‘unknown’ age status were found in reproductive condition in the year they were first encountered.

**Table 1:** Summary of top-ranked models based on model selection using AICc (see Supplementary Tables S2–S4). Model I = Sexual maturation in juvenile males. Model II = The influence of age and reproductive condition on body mass. Model III = Phenology of reproductive condition in relation to species and age.

Response variable:	Model I	Model II	Model III
	YN	Body mass (g)	ABCD
**Explanatory variable (SE):**			
(Intercept)	−1.33 (0.56)*	−2.78 (0.55)***	
SPP (Species) Mn (*M. nattereri*)	−2.23 (0.23)***	−0.52 (0.16)**	−3.38 (1.37)*
ASFC (April Suitable Foraging Conditions)	4.06 (1.57)**	1.74 (0.43)***	11.85 (3.09)***
DOTY (Day of the Year)		0.01 (0.00)***	0.23 (0.01)***
ACL_BS (Age Class and Breeding Status) ACL_BS (J_RC) (Juvenile in Reproductive Condition) ACL_BS (A_NB) (Adult Non-breeder) ACL_BS (A_RC) (Adult in Reproductive Condition)		1.19 (0.27)*** 1.37 (0.53)** 1.43 (0.19)***	
FA (Forearm size)		0.22 (0.01)***	
AGE (1–3 years old) AGE (≥4 years old)			12.58 (1.60)*** 17.22 (1.53)***
A|B B|C C|D			36.72 (2.26)*** 41.71 (2.39)*** 45.07 (2.49)***
SPP (Mn) * DOTY (Interaction)		−0.00 (0.00)***	−0.01 (0.01)
ASFC * DOTY (Interaction)		−0.01 (0.00)***	−0.06 (0.02)***
SPP (Mn) * ACL_BS (J_RC) (Interaction) SPP (Mn) * ACL_BS (A_NB) (Interaction) SPP (Mn) * ACL_BS (A_RC) (Interaction)		0.15 (0.11) −0.02 (0.33) −0.43 (0.07)***	
DOTY * ACL_BS (J_RC) (Interaction) DOTY * ACL_BS (A_NB) (Interaction) DOTY * ACL_BS (A_RC) (Interaction)		−0.01 (0.00)*** −0.00 (0.00) −0.00 (0.00)	
SPP (Mn) * AGE (1–3) (Interaction) SPP (Mn) * AGE (≥4) (Interaction)			1.37 (0.51)** 2.30 (0.55)***
DOTY * AGE (1–3) (Interaction) DOTY * AGE (≥4) (Interaction)			−0.06 (0.01)*** −0.08 (0.01)***
No. Observations	804	3065	1943
No. Years (fYR)	13 (0.51)	13 (0.01)	12 (0.41)
No. Individuals (IND)		1257 (0.12)	687 (0.80)
Residual variance		0.28	

*p < 0.05, **p < 0.01, ***p < 0.001. See Supplementary Tables S2–S4 for further details.

From one year of age onwards, almost all adult males (over 85% of *M. nattereri*, and more than 98% of *M. daubentonii*, respectively) came into reproductive condition each season (see Fig. 1a and Supplementary Table S1).

### Spring weather conditions affect the proportion of juvenile males in reproductive condition

**Figure 2:**
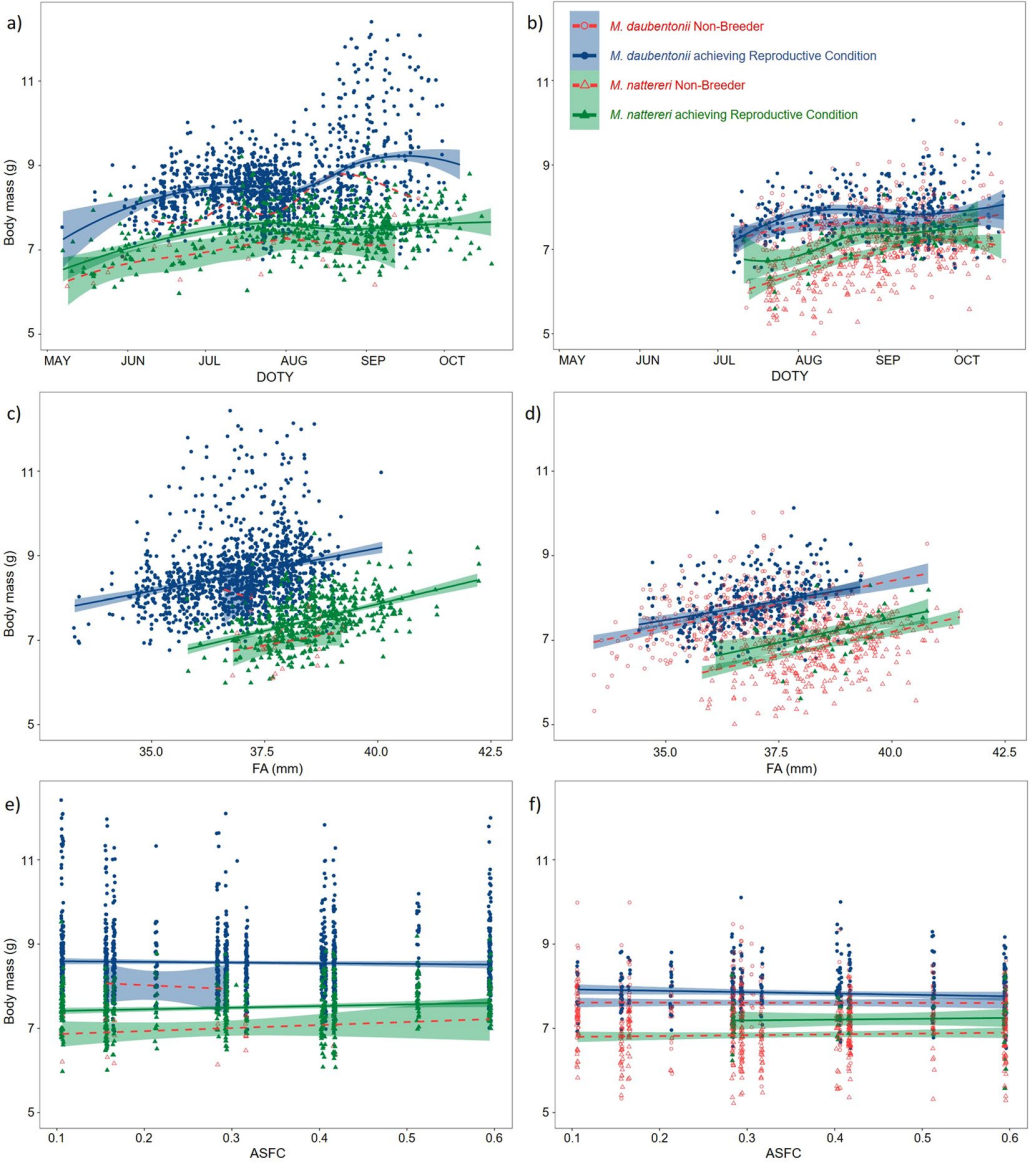
Body mass (g) of male *Myotis daubentonii* (circles) and *M. nattereri* (triangles) plotted for adult males (left panels; a, c and e) and juvenile males (right panels; b, d and f) against: DOTY (day of the year) (**a**,**b**), FA (forearm size (mm)) (**c**,**d**), and ASFC (an index of spring weather conditions) (**e**,**f**). Mean body mass is plotted (filled blue circles and solid blue lines for *M. daubentonii*, filled green triangles and solid green lines for *M. nattereri*, achieving reproductive condition that season. Red open symbols and dashed lines for non-breeders that season in both species), fit by loess (**a**,**b**) or linear regression (**c**–**f**), shaded areas (blue for *M. daubentonii*, and green for *M. nattereri*) represent 95% confidence intervals.

ASFC (April Suitable Foraging Conditions; our index of spring weather conditions) was retained during model selection as a significant predictor of the proportion of juvenile males becoming sexually mature each season (see Fig. 1b and Model I in Table 1). For *M. daubentonii* the proportion of juvenile males in reproductive condition in 2011 (the breeding season with the highest ASFC, and the earliest parturition dates, in our dataset) was 46/63 (73.02%), compared to only 7/43 (16.28%) in 2012 (the breeding season with the lowest ASFC, and latest parturition dates, in our dataset). Similarly, the majority of male *M. nattereri* juveniles that reached puberty were encountered during the five years with the highest ASFC values in our dataset, and no juvenile male *M. nattereri* were recorded as reaching sexual maturity during the four years with the lowest ASFC values in our dataset (see Fig. 2f).

### Body mass varies with age and reproductive condition

Juvenile non-breeders had lower body mass, on average, than males that became sexually mature as juveniles (see Fig. 2b and Model II in Table 1). Adult males that achieved reproductive condition were significantly heavier on average than adult non-breeders, and adults were significantly heavier on average than juveniles in both species (see Fig. 2 and Model II in Table 1). Body mass was also influenced by day of the year (DOTY), and interactions between DOTY and species (SPP), age class and reproductive condition (ACL_RC), and ASFC (see Fig. 2, and Model II in Table 1, and Supplementary Table S3). Forearm (FA) was retained in the top-ranked model as a significant predictor of male body mass (see Model II in Table 1). Male *M. daubentonii* are, on average, significantly heavier (but have smaller forearms) than male *M. nattereri* (see Fig. 2c,d).

### Phenology of reproductive condition in relation to species and age

**Figure 3:**
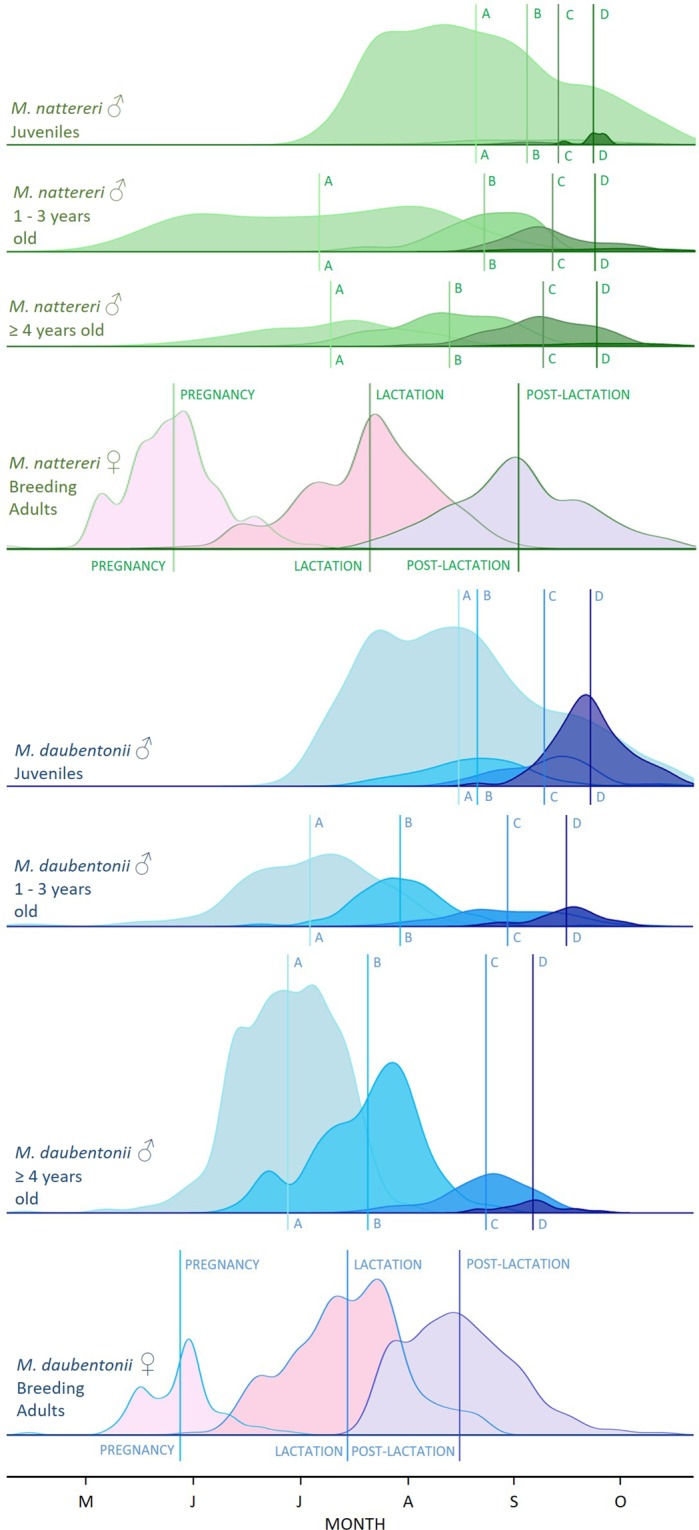
Phenology of phases of spermatogenesis (A > B > C > D) (see methods and supplementary Fig. S2), in male *M. nattereri* (green) and *M. daubentonii* (blue) per age class. Lines A–A, B–B, C–C, and D–D, indicate mean date for that reproductive phase per species and age class. The y-axis corresponds to count data (sample size). Female reproductive phenology is also presented (not to scale on y-axis) for temporal comparison.

The onset of spermatogenesis occurred earlier in male *M. daubentonii* compared to *M. nattereri* of equivalent age class (see Fig. 3 and Model III in Table 1). In both species the onset of spermatogenesis was advanced in older males (age ≥4 years old) compared to young adult males (aged 1–3 years old). Spermatogenesis occurred significantly later in juveniles than in adults in both species (see Fig. 3 and Model III in Table 1). An interaction between DOTY and ASFC was retained in the top-ranked model indicating that reproductive phenology was advanced during breeding seasons with a higher ASFC (see Model III in Table 1 and Supplementary Table S4).

## Discussion

As predicted, rates of sexual maturation in juvenile males, and the reproductive phenology of adult males, were found to correspond with the phenology of breeding females within our study system^14^. Earlier parturition dates in *M. daubentonii* compared to *M. nattereri* corresponded with higher proportions of juvenile male *M. daubentonii* than *M. nattereri* reaching puberty at 2–5 months old. During breeding seasons with favourable spring weather conditions, when parturition dates were earlier than average, higher proportions of juvenile males became sexually mature in both species. Males in breeding condition had higher body mass, on average, compared to males in the equivalent age class that were non-reproductive. Adult male *M. daubentonii* commenced spermatogenesis earlier than *M. nattereri*, and in both species older males were found to be more advanced in their reproductive condition compared to young adult males.

Adult *M. daubentonii* males typically commence spermatogenesis during June and July then leave our study system (summer habitat) when they become sexually active (with sperm stored in the epididymides indicating mating readiness^6,21^), presumably to participate in autumn swarming. Mating at swarming sites may maximise their breeding potential through copulation with numerous sexual partners and outbreeding with non-related females from different colonies drawn from a large catchment area^16^. In contrast, *M. nattereri* adult males typically commence spermatogenesis during July and August then leave their summer habitat (presumably to migrate to autumn swarming sites^23–25^) during September. These observations fit well with the temporal distribution of swarming activity recorded in these species, with the peak in *M. daubentonii* activity at swarming sites occurring during August, and the peak in *M. nattereri* activity at swarming sites occurring during September^25–28^. The difference in timing of spermatogenesis between species also corresponds with the breeding phenology of females, with independent juveniles and post-lactating *M. daubentonii* being recorded from early July onwards, whilst volant juveniles and post-lactating *M. nattereri* are recorded from late July onwards in our study system^14^.

Adult males in breeding condition (with sperm in their epididymides) have been found sharing day roosts with potentially receptive females from late July (*M. daubentonii*) and mid-August (*M. nattereri*) onwards on numerous occasions, suggesting that summer resident males may be responsible for the paternity of some juveniles within our study system^16,29^. In the absence of behavioural data on mating activities, or genetic data to assign paternity, there is currently no data available on whether or not juvenile males that become sexually mature are mating or fathering offspring.

Potential sampling bias exists within our dataset as males commencing spermatogenesis were recorded from June onwards whilst non-breeders were determined only during September or October (males encountered in non-reproductive condition during June to August may have commenced spermatogenesis later that breeding season when not observed). However, there is also a counter bias whereby males found to have commenced spermatogenesis during June to August are rarely encountered during September and October, probably due to their migration to autumn swarming and winter hibernation sites. The adult males that are recorded during September and October are more likely to be non-breeders, or males that commenced spermatogenesis later than average, which counter-balances to some extent the longer time period over which males in reproductive condition are recorded. From mid-September onwards the majority of males encountered within this summer habitat are juveniles (see Fig. 3).

Spermatogenesis may be triggered by environmental or endogenous cues^30^, and selection pressure may operate to synchronise the energy expenditure associated with spermatogenesis to seasonal peaks in food availability^2,18^, rather than there being any direct synchronisation with female reproductive cycles (except for juvenile males which are likely to be directly influenced by parturition dates and consequent developmental and maturation rates). Nevertheless, despite the temporal separation between spermatogenesis and mating that is possible in the reproductive cycle of temperate bats due to prolonged sperm storage^5,10^ it seems likely that a selective advantage does exist for males to be ready to mate soon after females have completed lactation. Mating within day roosts in the summer habitat^16^, and peak autumn swarming activities^25–28^, both occur within a short period that is post-maternity and pre-hibernation for our two study species. The timing of mating readiness in males and receptivity in females differs between these sympatric congeners but is highly synchronised within each species. The ‘early male advantage’ hypothesis^6,31^ is also supported by our empirical evidence that older males (which are in better body condition, see Supplementary Fig. S1), commence spermatogenesis and are in breeding condition earlier than young adult or juvenile males.

Whether or not selection pressure exists for early maturation in juvenile males remains uncertain. The fact males that undergo spermatogenesis as juveniles have higher body mass, on average, than juveniles which remain immature, suggests early maturation is not a costly trait given suitable conditions (e.g. early birth timing which allows sufficient time for development and puberty prior to hibernation). The proportion of juvenile males mating successfully remains unknown. Even low incidence of paternity amongst juveniles could significantly contribute to lifetime reproductive success at the individual level, and affect gene flow at the population level, as survival rates are lower in juveniles than adults^32^.

Although forearm size was a significant predictor of body mass, using a body condition index to account for variation in body size based on forearm measurements would not necessarily provide a better predictor of fat mass than uncorrected body mass^33^. Body mass has been empirically proven to be significantly positively correlated with fat mass in *Myotis lucifugus*^31^, a Nearctic species with morphological similarities to the Palearctic *M. daubentonii*^34^. Previous studies^31^ have also noted a small proportion of exceedingly heavy (>10.0 g) adult males. We observe that such heavyweights are predominantly older male *M. daubentonii*, especially during mid-August to mid-September (see Supplementary Fig. S1). Due to the energetic constraints imposed by flight it is deemed unlikely that such heavyweight males participate in swarming for their main mating strategy (heavyweight males are indeed underrepresented in swarming captures, DML unpublished data). It therefore seems likely that several, not entirely mutually exclusive, and possibly varying with age and body condition, mating strategies exist. These include fathering offspring with females from maternity (even natal) colonies resident in the same summer habitat^16,29^, autumn swarming^16,24^, and a third possibility, that heavyweight males are able to sustain more frequent arousals and copulations during hibernation^8^. Our findings correspond with previous observations^19^ that although male bats are capable of reaching sexual maturity at 2–5 months of age, full maturation (i.e. earlier onset of spermatogenesis, and possibly a shift in mating strategies, with increasing age) is a process that takes several years. Demonstrating that, as small-sized but long-lived mammals, bats exhibit unusual combinations of life-history traits^35^.

Our results also indicate that using sexual immaturity as a determining factor for distinguishing juvenile males during autumn is not valid for all medium-sized temperate *Myotis* species^6–8,31,36^. Altitudinal and latitudinal gradients, however, may contribute to geographical variation in the proportions of juveniles reaching puberty due to variation in the timing of parturition (in relation to severity of spring climate) and therefore time available to complete development prior to hibernation^20,31,37^.

Many temperate zone bat species exhibit adaptations to incorporate hibernation within their reproductive cycle, including an extended mating season lasting from late summer into autumn, through winter, and even into spring^6,9,16,31^. In many species both sexes are capable of storing sperm for up to several months^4^, which enables inseminated females to ovulate and commence gestation with minimal energy expenditure in spring^10,11^. These adaptations may make hibernating bats less sensitive to mismatches in phenology and climatic variation than other taxa where all reproductive stages occur in rapid succession within a restricted period during which breeding success is dependent on sustained favourable conditions^1^.

## Methods

### Data collection

Bats were encountered in day roosts within Schwegler^TM^ woodcrete boxes distributed across our study site, Wytham Woods (51°77′N, 1°33′W) or caught at night in mist nets and harp traps in the same locality. All catching, handling and ringing of bats was carried out under project licence from Natural England (2018-36143-SCI-SCI and preceding licences). All bats were returned to their roost, or released at the site of capture, as soon as possible following examination.

### Assessment of reproductive condition

The presence or absence of enlarged testes and distended caudae epididymides (swelling being indicative of spermatogenesis and sperm storage respectively), was recorded based on visual assessment by experienced surveyors during manual handling of bats.

Males were classified into four categories of reproductive condition^38^: (A) non-reproductive (testes not swollen, epididymides not distended), (B) testes swollen, epididymides not distended, (C) testes swollen, epididymides distended, and D) testes not swollen, epididymides distended, see Supplementary Fig. S2). In male bats there is a progression from A > B > C > D (a process taking 1–2 months per individual once commenced, based on 271 within-season recaptures of 105 males that were observed to progress through stages of spermatogenesis) per breeding season in all reproductively active males.

Assessment of whether or not individual males were in reproductive condition each season was based on any encounter where swollen testes and/or epididymides (reproductive condition B, C or D) were observed during June to October. Encounters with adult males with distended epididymides but without enlarged testes in spring (n = 27 bats on 30 occasions during April and May) were excluded in an attempt to record the temporal occurrence of spermatogenesis per annum (i.e. enlargement of the testes due to spermatogenesis prior to distension of the caudae epididymides due to sperm storage), and not vestiges of the previous breeding season. Only males encountered during September or October in non-reproductive condition (A) were classified as non-breeders that season. Individuals encountered only during April to August in non-reproductive condition (A) were classed as ‘unknown’ breeding status for that season, as they might still have come into reproductive condition later whilst not observed. Some individuals found in non-reproductive condition (A) during August were recaptured in reproductive condition (B or C) during September.

### Age assessment

Males were classified as juvenile (actual age = 0) based on unfused finger joints^39^ or a suite of secondary characteristics indicative of bats born that season, including ‘fresh’ wing membranes, grey pelage, dense ‘chinspot’, dark tunica vaginalis, and light weight^18,32,40,41^, once the epiphyseal gaps were fully ossified. Adult males (minimum age = 1) were identified on first capture based on timing (encountered early in the season before juveniles were full-grown), evidence of prior reproduction such as elongation of the caudae epididymides or unpigmented tunica vaginalis, and colouration or weight^15^. Bats of indeterminate age, possessing an ambiguous mixture of juvenile and adult characteristics, were classified as ‘unknown’ (minimum age = 0) when ringed. The majority of data for adult males (2324/2693 = 86.30% of *M. daubentonii* encounters, and 807/887 = 90.98% of *M. nattereri* encounters) were derived from confirmed adults (i.e. ringed individuals recaptured 1–12 years post-ringing).

The proportion of males in reproductive condition per age class was based only on individuals of known (actual) age for age classes 0, 1, 2 and 3 (ringed as juveniles and recaptured 0–3 years later), age class ≥4 included bats of known and minimum age (ringed at least four years before as a juvenile or ‘unknown’, or at least three years earlier as an adult).

### Biometric measurements

Forearm length was measured to the nearest 0.1 mm using dial calipers (n = 3779, 1–24 measurements per male). Body mass was recorded to the nearest 0.1 g using a spring balance (n = 3598, 1–22 measurements per male). Body mass measurements obtained from males captured at night were excluded from our analyses as during foraging bouts substantial weight gain can occur due to ingested prey.

### Data Analysis

#### Model I: Reproductive condition in juvenile males

The proportion of juvenile males reaching puberty was treated as a binary response variable (Y = found in reproductive condition (B, C or D) as a juvenile, N = found in non-reproductive condition (A) during September or October in the year of their birth) in logistic models, using the ‘glmer’ function in package lme4^42^ in R version 3.5.3^43^, with species (SPP = *Myotis daubentonii*, abbreviated to Md, or *M. nattereri*, abbreviated to Mn) as a categorical explanatory variable, and spring weather conditions during their birth year (ASFC which is a proxy for breeding phenology and birth timing^14^) as a continuous explanatory variable. We also tested for an interaction between SPP:ASFC in case the effect of spring phenology varied between species (details of model selection are presented in Supplementary Table S2). Year as a factor (fYR) was included as a random intercept effect to provide the correct level of replication.

#### Model II: Does body mass vary with age and reproductive condition?

Due to the temporal spread of body mass (g) and reproductive condition (A, B, C and D) data throughout the summer survey season (see Figs. 2a,b and 3) it was not possible to incorporate weight as an explanatory variable in an analysis of reproductive condition as determining cause and effect between body mass and reproductive condition was problematic (i.e. the weight of males in non-reproductive condition A may influence the likelihood and timing of spermatogenesis, but the body mass of males in reproductive condition B and C may have been influenced by energy expenditure during spermatogenesis). We therefore used body mass as the response variable in linear mixed models, using the ‘lmer’ function in R package lme4^42^, with SPP, and age class and breeding status (ACL_BS with four categories; J_NB = juvenile non-breeder, J_RC = juvenile achieving puberty, A_NB = adult non-breeder that season, and A_RC = adult in reproductive condition that season) as categorical explanatory variables, and day of the year (DOTY), ASFC and forearm size (FA) as continuous explanatory variables. We also tested for interactions between predictor variables in case the effect of ACL_BS varied between SPP, or in case the effects of DOTY and ASFC varied between SPP, ACL_BS, or in relation to FA, an interaction between DOTY and ASFC was also tested (see Supplementary Table S3 for details of model selection). Because these datasets contained repeat measures (individuals were observed on between 1–8 occasions per annum, for 1–13 breeding seasons) both individual (IND = ring no. a unique identifier per individual) and fYR were included as random intercept effects to give the correct level of replication. Based on recent recommendations^33^ we did not correct body mass for forearm size. Due to visual examination of Fig. 2c,d, however, FA was included as a predictor variable.

#### Model III: Phenology of reproductive condition in relation to species and age

Cumulative Link Mixed Models (CLMMs) with the progression of reproductive condition (A > B > C > D) on an ordinal scale as our response variable were performed using the ‘clmm’ function in package ordinal^44^ in R. SPP and age class (ACL with three categories; juveniles, young adults (aged 1–3 years), and older males (aged ≥4 years old), were included as categorical explanatory variables. DOTY and ASFC were included as continuous explanatory variables. We tested for differences in the temporal distribution of reproductive phases by including interaction terms between DOTY, SPP and ACL. We also tested for interactions between ASFC and the other predictor variables (see Supplementary Table S4 for details of model selection). IND and fYR were included as random intercept effects to provide the correct level of replication. Only bats known to have commenced spermatogenesis that breeding season were included in the CLMM dataset.

#### Model selection

Akaike Information Criterion corrected for small sample size (AICc) and model weights were derived using package AICcmodavg^45^ in R, and were used to rank model sets (details of model selection are provided in Supplementary Tables S2–S4) in accordance with an Information-Theoretic approach^46^. Results from the favoured model in each set are presented in Table 1.

### Ethical approval

All catching, handling and ringing of bats was carried out under project licence from Natural England (2018-36143-SCI-SCI and preceding licences). These methods were carried out in accordance with relevant international, national and institutional guidelines and regulations.

## Supplementary information

Supplementary Information

## Data Availability

Data is available from the corresponding author on request.
