# Supplementary material for: Phenology of reproductive condition varies with age and spring weather conditions in male Myotis daubentonii and M. nattereri (Chiroptera: Vespertilionidae)
Source: Sci Rep. 2020 Apr 20;10:6664. doi: 10.1038/s41598-020-63538-y (PMC7171103; doi:10.1038/s41598-020-63538-y)
Supplement: Supplementary file 1 — Supplementary Information [file 41598_2020_63538_MOESM1_ESM.pdf]

SUPPLEMENTARY INFORMATION: Phenology of reproductive condition varies with age and spring weather conditions in male *Myotis daubentonii* and *M. nattereri* (Chiroptera: Vespertilionidae).

Danielle M. Linton (corresponding author: daniellemlinton@gmail.com) and David W. Macdonald

Table S1 Proportion of male bats in reproductive condition per species (Md = *Myotis daubentonii*, Mn = *M. nattereri*), and age class (0, 1, 2, 3, and ≥4). n = sample size (minimum number alive), Y = bats found with swollen testes and/or distended epididymides (reproductive condition B, C or D), N = bats found without swollen testes or distended epididymides (non-reproductive condition A) during September and/or October, and U = unknown (bats not observed that year, or bats not in reproductive condition during June to August and not observed during September or October).

Species	Age Class	n	Y	N	U	Y / (Y + N)	N / (Y + N)	(Y + N) / n	U / n
Md	0	931	282	273	376	0.508	0.492	0.596	0.404
Md	1	256	106	2	148	0.981	0.019	0.422	0.578
Md	2	146	46	1	99	0.979	0.021	0.322	0.678
Md	3	99	35	0	64	1	0	0.354	0.646
Md	≥4[1]	1170	402	0	768	1	0	0.344	0.656
Mn	0	397	32	217	148	0.129	0.871	0.627	0.373
Mn	1	169	86	13	70	0.869	0.131	0.586	0.414
Mn	2	83	42	3	38	0.933	0.067	0.542	0.458
Mn	3	41	25	1	15	0.962	0.038	0.634	0.366
Mn	≥4[1]	141	82	0	59	1	0	0.582	0.418

[1] Bats of known age and minimum age (based on ring capture history and age when ringed).

Table S2 Model I: Reproductive condition in juvenile male bats. Results of model selection based on Akaike Information Criterion corrected for small sample size (AICc) for Generalized Linear Mixed Models (GLMMs) with sexual maturation as a binary response variable (Y = 1, N = 0), species (SPP = *M. daubentonii* or *M. nattereri*) as a categorical explanatory variable, Index of Spring Foraging Conditions (ASFC) as a continuous explanatory variable, and year as a factor (fYR) as a random intercept effect. Model fit by maximum likelihood (Laplace Approximation) with binomial error distribution and logit link function. **Favoured model (simpler nested model within 2 ΔAICc units of the top ranked model) in bold.** Dataset comprises 804 observations (of 804 juvenile male individuals) across thirteen breeding seasons (2006 - 2018).

Model structure	*df*	Residual Deviance	K	AICc	ΔAICc	w_i	logLik
YN ~ SPP * ASFC + (1|fYR)	799	891.3	5	901.41	0.00	0.48	-445.67
**YN ~ SPP + ASFC + (1|fYR)**	**800**	**893.5**	**4**	**901.58**	**0.17**	**0.44**	**-446.77**
YN ~ SPP + (1|fYR)	801	898.8	3	904.88	3.46	0.08	-449.42
YN ~ ASFC + (1|fYR)	801	1023.2	3	1029.20	127.79	0.00	-511.59
YN ~ (1|fYR)	802	1027.7	2	1031.74	130.33	0.00	-513.86

df = degrees of freedom, K = number of parameters, W_i = Akaike weight, logLik = loglikelihood.

Table S3 Model II: The influence of species, age and breeding status, date, spring weather conditions, and forearm size on male body weights. Results of model selection for linear mixed models fit by maximum likelihood with body mass (W = weight(g)) as the response variable, species (SPP = *M. daubentonii* or *M. nattereri*), and age class and breeding status (ACL_BS with 4 categories: J_NB = juvenile non-breeder, J_RC = juvenile achieving reproductive condition, A_NB = adult non-breeder, and A_RC = adult achieving reproductive condition that season) as categorical explanatory variables, day of the year (DOTY), Index of Spring Foraging Conditions (ASFC) and forearm size (FA (mm)) as continuous explanatory variables, and individual (IND), and year as a factor (fYR) as random intercept effects. Dataset comprises 3065 observations from 1257 individuals across 13 breeding seasons (2006 - 2018).

Model structure + (1|IND) + (1|fYR)	K	AICc	ΔAICc	W_i	logLik
**W ~ SPP * ACL_BS + DOTY * ASFC + FA + SPP:DOTY + ACL_BS:DOTY**	**19**	**5648.52**	**0.00**	**0.99**	**-2805.14**
W ~ SPP * ACL_BS + DOTY * ASFC + FA + SPP:DOTY	16	5659.29	10.77	0.01	-2813.56
W ~ SPP * ACL_BS + DOTY * ASFC + FA + ACL_BS:DOTY	18	5663.81	15.28	0.00	-2813.79
W ~ SPP * ACL_BS + DOTY * ASFC + FA	15	5669.42	20.90	0.00	-2819.63
W ~ SPP * ACL_BS + DOTY + ASFC + FA + ACL_BS:DOTY	17	5680.87	32.34	0.00	-2823.33
W ~ SPP * ACL_BS + DOTY + FA	13	5687.44	38.92	0.00	-2830.66
W ~ SPP * ACL_BS + DOTY + ASFC + FA + ACL_BS:ASFC	17	5689.25	40.73	0.00	-2827.53
W ~ SPP * ACL_BS + DOTY + ASFC + FA	14	5689.45	40.93	0.00	-2830.65
W ~ SPP * ACL_BS + DOTY * FA + ASFC	15	5690.02	41.50	0.00	-2829.93
W ~ SPP * ACL_BS + DOTY * FA	15	5690.02	41.50	0.00	-2829.93
W ~ SPP * ACL_BS + DOTY + ASFC * FA	15	5690.94	42.41	0.00	-2830.39
W ~ SPP * ACL_BS + DOTY + FA + ACL_BS:FA	16	5692.16	43.64	0.00	-2829.99
W ~ SPP * ACL_BS + DOTY + ASFC + FA + ACL_BS:FA	17	5694.18	45.66	0.00	-2829.99
W ~ SPP * ASFC + ACL_BS + DOTY + FA	12	5705.93	57.41	0.00	-2840.91
W ~ SPP + ACL_BS + DOTY * ASFC + FA	12	5709.23	60.71	0.00	-2842.57
W ~ SPP + ACL_BS * FA + DOTY	13	5716.03	67.51	0.00	-2844.96
W ~ SPP + ACL_BS * DOTY + FA	13	5717.49	68.96	0.00	-2845.68
W ~ SPP + ACL_BS * FA + DOTY + ASFC	14	5718.05	69.53	0.00	-2844.96
W ~ SPP + ACL_BS * DOTY + ASFC + FA	14	5719.5	70.98	0.00	-2845.68
W ~ SPP + ACL_BS * DOTY + ASFC + FA + ACL_BS:ASFC	17	5719.84	71.32	0.00	-2842.82
W ~ SPP + ACL_BS + DOTY + FA	10	5724.05	75.52	0.00	-2851.99
W ~ SPP * DOTY + ACL_BS + FA	11	5724.51	75.98	0.00	-2851.21
W ~ SPP * FA + ACL_BS + DOTY	11	5726.01	77.48	0.00	-2851.96
W ~ SPP + ACL_BS + DOTY + ASFC + FA	11	5726.06	77.53	0.00	-2851.99
W ~ SPP + DOTY * FA + ACL_BS	11	5726.06	77.54	0.00	-2851.99
W ~ SPP + ACL_BS * ASFC + DOTY + FA	14	5726.25	77.73	0.00	-2849.06
W ~ SPP * DOTY + ACL_BS + ASFC + FA	12	5726.52	78.00	0.00	-2851.21
W ~ SPP + ACL_BS + DOTY + ASFC * FA	12	5726.53	78.01	0.00	-2851.21
W ~ SPP * DOTY + ACL_BS * ASFC + FA	15	5726.85	78.33	0.00	-2848.35
W ~ SPP * FA + ACL_BS + DOTY + ASFC	12	5728.02	79.50	0.00	-2851.96
W ~ SPP + ACL_BS + DOTY * FA + ASFC	12	5728.07	79.55	0.00	-2851.98
W ~ SPP * ACL_BS + DOTY * ASFC + ACL_BS:DOTY + SPP:DOTY	18	5873.09	224.57	0.00	-2918.43
W ~ SPP * ACL_BS + DOTY * ASFC + SPP:DOTY	15	5885.00	236.48	0.00	-2927.42

W ~ SPP * ACL_BS + DOTY * ASFC + ACL_BS:DOTY	17	5888.15	239.62	0.00	-2926.97
W ~ SPP * ACL_BS + DOTY * ASFC	14	5894.95	246.43	0.00	-2933.41
W ~ SPP * ACL_BS + DOTY + ASFC + ACL_BS:DOTY	16	5904.99	256.47	0.00	-2936.41
W ~ SPP * ACL_BS + DOTY	12	5912.34	263.81	0.00	-2944.12
W ~ SPP * ACL_BS + DOTY + ASFC	13	5914.29	265.77	0.00	-2944.09
W ~ SPP * ACL_BS + DOTY + ASFC + ACL_BS:ASFC	16	5915.85	267.33	0.00	-2941.83
W ~ SPP * ASFC + ACL_BS + DOTY	11	5939.98	291.45	0.00	-2958.95
W ~ SPP + ACL_BS + ASFC * DOTY	11	5941.01	292.49	0.00	-2959.46
W ~ SPP + ACL_BS * DOTY	12	5948.78	300.26	0.00	-2962.34
W ~ SPP + ACL_BS * DOTY + ASFC	13	5950.76	302.24	0.00	-2962.32
W ~ SPP + ACL_BS * DOTY + ASFC + ACL_BS:ASFC	16	5952.52	304.00	0.00	-2960.17
W ~ SPP + ACL_BS + DOTY	9	5955.23	306.71	0.00	-2968.58
W ~ SPP * DOTY + ACL_BS	10	5956.09	307.57	0.00	-2968.01
W ~ SPP + ACL_BS + DOTY + ASFC	10	5957.20	308.68	0.00	-2968.56
W ~ SPP * DOTY + ACL_BS + ASFC	11	5958.06	309.53	0.00	-2967.98
W ~ SPP + ACL_BS * ASFC + DOTY	13	5958.69	310.16	0.00	-2966.28
W ~ SPP * DOTY + ACL_BS * ASFC	14	5959.65	311.13	0.00	-2965.76
W ~ SPP * ACL_BS + FA	12	5994.28	345.75	0.00	-2985.09
W ~ SPP * ASFC + ACL_BS + FA	11	5996.03	347.51	0.00	-2986.97
W ~ SPP * ACL_BS + ASFC + FA	13	5996.28	347.76	0.00	-2985.08
W ~ SPP * ACL_BS + ASFC * FA	14	5997.58	349.06	0.00	-2984.72
W ~ SPP * ACL_BS + ASFC + FA + ACL_BS:ASFC	16	5997.82	349.30	0.00	-2982.82
W ~ SPP * ACL_BS + FA + ACL_BS:FA	15	5998.56	350.04	0.00	-2984.20
W ~ SPP * ACL_BS + ASFC + FA + ACL_BS:FA	16	6000.57	352.05	0.00	-2984.20
W ~ SPP + ACL_BS * FA	12	6007.58	359.05	0.00	-2991.74
W ~ SPP + ACL_BS + FA	9	6008.94	360.41	0.00	-2995.44
W ~ SPP + ACL_BS * FA + ASFC	13	6009.59	361.07	0.00	-2991.73
W ~ SPP * FA + ACL_BS	10	6010.82	362.30	0.00	-2995.37
W ~ SPP + ACL_BS + ASFC + FA	10	6010.94	362.42	0.00	-2995.43
W ~ SPP + ASFC * FA + ACL_BS	11	6011.44	362.92	0.00	-2994.68
W ~ SPP * FA + ACL_BS + ASFC	11	6012.83	364.31	0.00	-2995.37
W ~ SPP + ACL_BS * ASFC + FA	13	6013.24	364.72	0.00	-2993.56
W ~ SPP * ACL_BS	11	6230.41	581.89	0.00	-3104.16
W ~ SPP * ACL_BS + ASFC	12	6232.36	583.84	0.00	-3104.13
W ~ SPP * ACL_BS + ASFC + ACL_BS:ASFC	15	6235.58	587.06	0.00	-3102.71
W ~ SPP * ASFC + ACL_BS	10	6239.00	590.48	0.00	-3109.47
W ~ SPP + ACL_BS	8	6249.54	601.02	0.00	-3116.75
W ~ SPP + ACL_BS + ASFC	9	6251.49	602.97	0.00	-3116.72
W ~ SPP + ACL_BS * ASFC	12	6255.06	606.54	0.00	-3115.48
W ~ SPP + ASFC * DOTY + FA	9	6474.78	826.25	0.00	-3228.36
W ~ SPP * ASFC + DOTY + FA	9	6476.90	828.38	0.00	-3229.42
W ~ SPP + DOTY * FA	8	6486.51	837.99	0.00	-3235.23
W ~ SPP + DOTY + FA	7	6486.66	838.14	0.00	-3236.31
W ~ SPP + DOTY * FA + ASFC	9	6487.10	838.57	0.00	-3234.52
W ~ SPP + ASFC + DOTY + FA	8	6487.21	838.69	0.00	-3235.58
W ~ SPP * FA + DOTY	8	6487.94	839.42	0.00	-3235.95
W ~ SPP * DOTY + FA	8	6487.96	839.44	0.00	-3235.96
W ~ SPP * FA + ASFC + DOTY	9	6488.49	839.97	0.00	-3235.22
W ~ SPP + DOTY + ASFC * FA	9	6488.50	839.97	0.00	-3235.22
W ~ SPP * DOTY + ASFC + FA	9	6488.54	840.02	0.00	-3235.24
W ~ SPP * ASFC + FA	8	6532.90	884.38	0.00	-3258.43

W ~ SPP + FA	6	6541.11	892.59	0.00	-3264.54
W ~ SPP + ASFC + FA	7	6541.93	893.41	0.00	-3263.95
W ~ SPP * FA	7	6542.83	894.31	0.00	-3264.40
W ~ SPP + ASFC * FA	8	6543.20	894.68	0.00	-3263.58
W ~ SPP * FA + ASFC	8	6543.65	895.13	0.00	-3263.80
W ~ SPP + ASFC * DOTY	8	6691.99	1043.47	0.00	-3337.97
W ~ SPP * ASFC + DOTY	8	6694.85	1046.33	0.00	-3339.40
W ~ SPP + DOTY	6	6703.34	1054.82	0.00	-3345.66
W ~ SPP + DOTY + ASFC	7	6703.69	1055.16	0.00	-3344.82
W ~ SPP * DOTY	7	6705.08	1056.55	0.00	-3345.52
W ~ SPP * DOTY + ASFC	8	6705.43	1056.91	0.00	-3344.69
W ~ SPP * ASFC	7	6765.76	1117.24	0.00	-3375.86
W ~ SPP	5	6772.75	1124.23	0.00	-3381.37
W ~ SPP + ASFC	6	6773.15	1124.62	0.00	-3380.56

AICc = Akaike Information Criterion (corrected), K = number of parameters, W_i = Akaike weight.

Figure S1. Seasonal distribution of body mass (g), in adult male *Myotis daubentonii* (MD, blue circles), and *M. nattereri* (MN, green triangles), discriminating between young adults (YA, aged 1 - 3 years old, open symbols) and older males (OM, ≥4 years old, filled symbols). Mean body mass, with 95% confidence interval (shaded areas), for young adults (dashed line) and older males (solid line) fit by loess regression in R (function geom_smooth() using ggplot2).

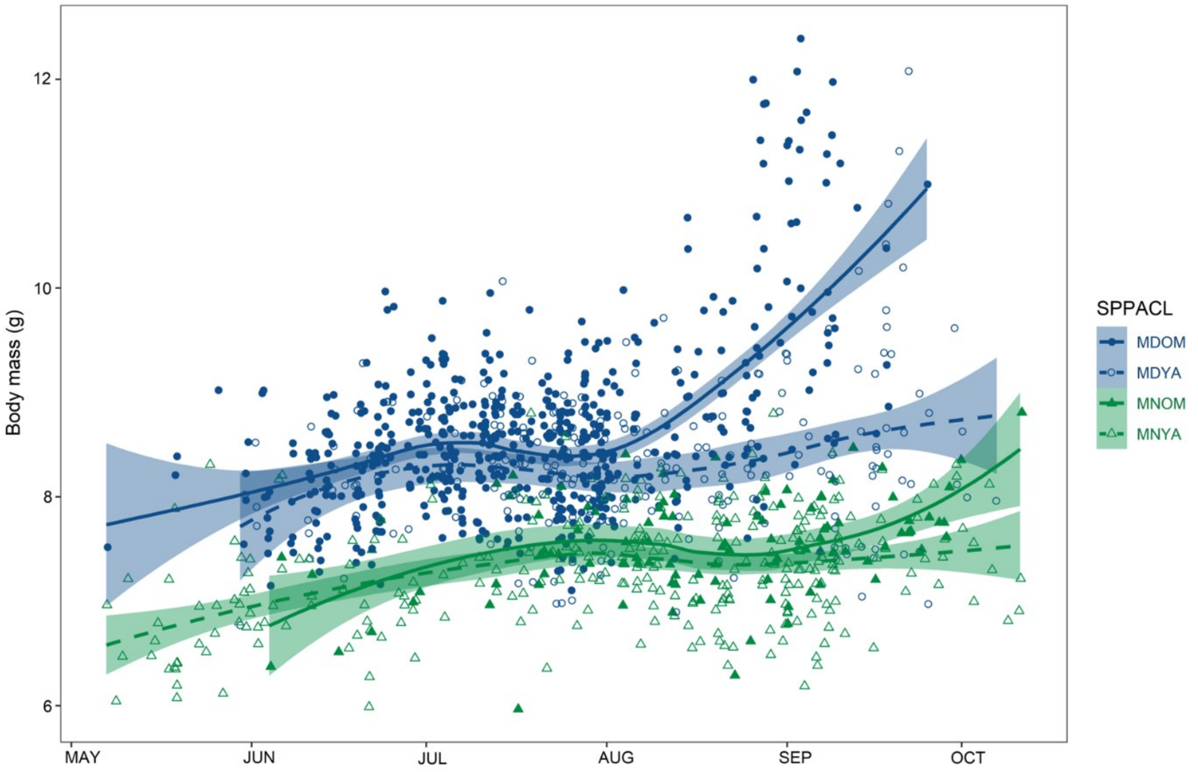

Figure S2. Reproductive condition in male bats: A = testes not swollen, epididymides not distended (juvenile bat with pigmented tunica vaginalis), B = testes swollen, epididymides not distended (adult bat with elongated caudae epididymides), C = testes swollen, epididymides distended, and D = testes not swollen, epididymides distended.

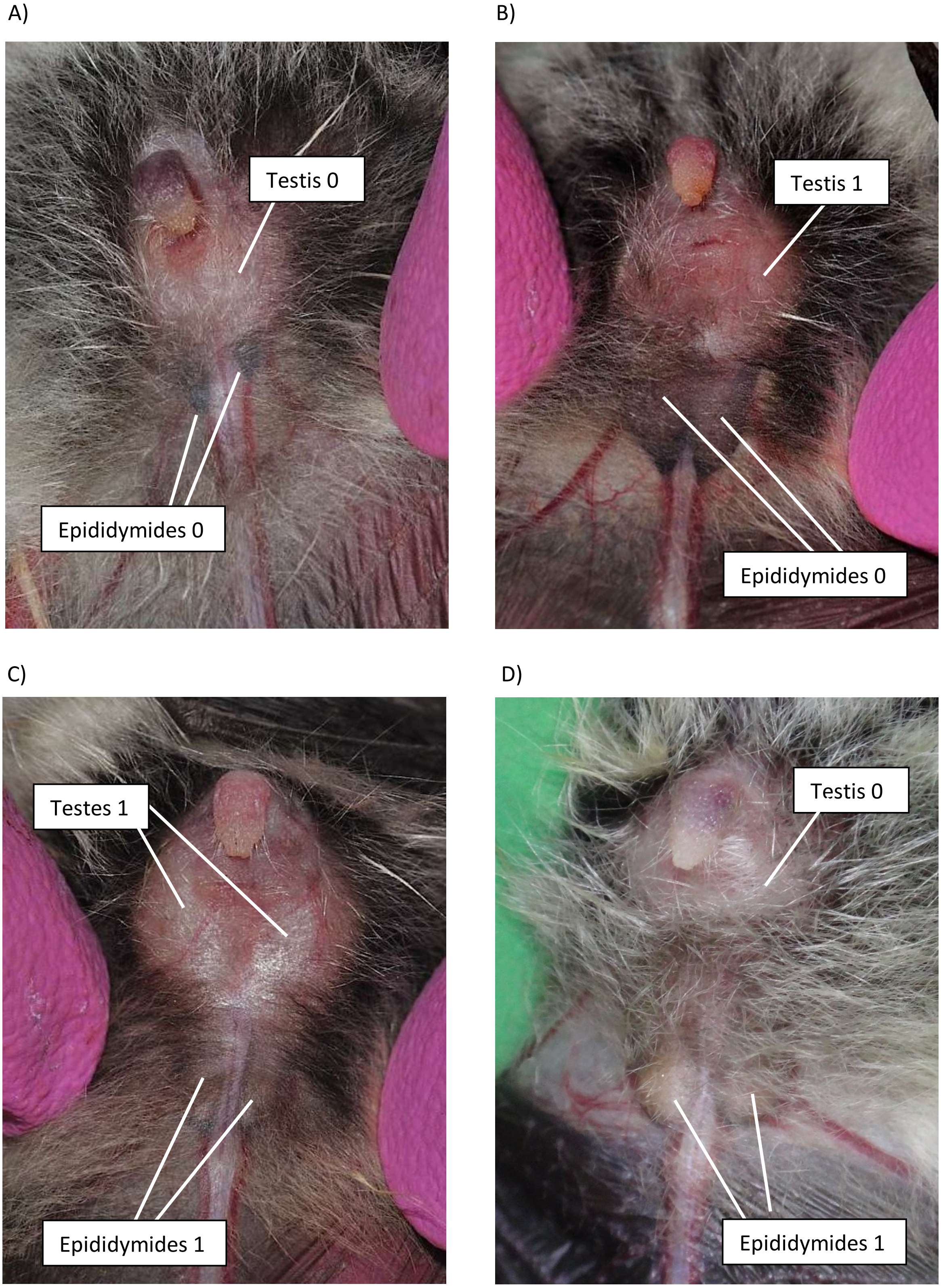

Table S4 Model III: Phenology of male reproductive condition and the influence of species, age class and spring weather conditions on timing of spermatogenesis and distension of the caudae epididymides. Results of model selection based on Akaike Information Criterion corrected for small sample size (AICc) for Cumulative Link Mixed Models (CLMMs) with reproductive condition on an ordinal scale (A>B>C>D where A = testes not swollen, epididymides not distended, B = testes swollen, epididymides not distended, C = testes swollen, epididymides distended, and D = testes not swollen, epididymides distended) as the response variable, age class (ACL with 3 categories = juveniles, males aged 1 - 3 years old, and bats aged 4 years or older, based on ring capture histories and age when ringed) as a categorical explanatory variable, day of the year (DOTY), and Index of Spring Foraging Conditions (ASFC) as continuous explanatory variables, and individual (IND) and year as a factor (fYR) as random intercept effects. Model fit by maximum likelihood (Laplace Approximation) and logit link function. Dataset comprises 1943 observations from 687 individuals across 12 breeding seasons (2006 - 2017).

Model structure + (1|IND) + (1|fYR)	K	AICc	ΔAICc	W_i	logLik
**ABCD ~ DOTY * SPP + ACL + ASFC + DOTY:ACL + SPP:ACL + DOTY:ASFC**	**16**	**2431.77**	**0.00**	**1**	**-1199.74**
ABCD ~ DOTY * SPP + ACL + ASFC + DOTY:ACL + SPP:ACL	15	2445.24	13.46	0.00	-1207.49
ABCD ~ DOTY * SPP + ACL + ASFC + DOTY:ACL + DOTY:ASFC	14	2447.02	15.25	0.00	-1209.40
ABCD ~ DOTY * SPP + ACL + ASFC + DOTY:ACL + SPP:ACL + ACL:ASFC	17	2447.06	15.29	0.00	-1206.37
ABCD ~ DOTY * SPP + ACL + ASFC + DOTY:ACL + SPP:ACL + SPP:ASFC	16	2447.22	15.44	0.00	-1207.47
ABCD ~ DOTY * SPP + ACL + DOTY:ACL	12	2460.49	28.72	0.00	-1218.16
ABCD ~ DOTY * SPP + ACL + ASFC + DOTY:ACL	13	2462.10	30.33	0.00	-1217.96
ABCD ~ DOTY * SPP + ACL + ASFC + DOTY:ACL + SPP:ASFC	14	2462.25	30.48	0.00	-1217.02
ABCD ~ DOTY * ACL + SPP	11	2462.34	30.57	0.00	-1220.10
ABCD ~ DOTY * ACL + SPP + ASFC	12	2463.96	32.18	0.00	-1219.90
ABCD ~ DOTY * SPP + ACL + ASFC + DOTY:ACL + ACL:ASFC	15	2463.99	32.22	0.00	-1216.87
ABCD ~ DOTY * SPP + ACL + SPP:ACL	12	2552.07	120.30	0.00	-1263.96
ABCD ~ DOTY * SPP + ACL + ASFC + SPP:ACL + DOTY:ASFC	14	2552.91	121.14	0.00	-1262.35
ABCD ~ DOTY * SPP + ACL + ASFC + SPP:ACL	13	2553.15	121.38	0.00	-1263.48
ABCD ~ DOTY * SPP + ACL + ASFC + SPP:ACL + ACL:ASFC	15	2553.20	121.42	0.00	-1261.47
ABCD ~ DOTY + SPP * ACL	11	2554.78	123.01	0.00	-1266.32
ABCD ~ DOTY * SPP + ACL + ASFC + SPP:ASFC + SPP:ASFC	14	2554.97	123.20	0.00	-1263.38
ABCD ~ DOTY + SPP * ACL + ASFC	12	2555.84	124.07	0.00	-1265.84
ABCD ~ DOTY * SPP + ACL	10	2556.75	124.98	0.00	-1268.32
ABCD ~ DOTY * SPP + ACL + ASFC	11	2557.92	126.15	0.00	-1267.89
ABCD ~ DOTY * ASFC + SPP + ACL	11	2561.77	130.00	0.00	-1269.82
ABCD ~ DOTY + SPP + ACL	9	2561.90	130.13	0.00	-1271.90
ABCD ~ DOTY + SPP + ACL + ASFC	10	2563.07	131.30	0.00	-1271.48
ABCD ~ DOTY + SPP * ASFC + ACL	11	2563.27	131.50	0.00	-1270.57
ABCD ~ DOTY + SPP + ACL * ASFC	12	2563.48	131.70	0.00	-1269.66

K = number of parameters, W_i = Akaike weight, logLik = loglikelihood.
